# Quality control of malaria microscopy reveals misdiagnosed non-falciparum species and other microscopically detectable pathogens in Senegal

**DOI:** 10.1186/s12941-018-0261-1

**Published:** 2018-03-15

**Authors:** Mamadou Alpha Diallo, Khadim Diongue, Mame Cheikh Seck, Mouhamadou Ndiaye, Ibrahima Diallo, Younouss Diedhiou, Tolla Ndiaye, Yaye Die Ndiaye, Aida Sadikh Badiane, Daouda Ndiaye

**Affiliations:** 1 Laboratory of Parasitology and Mycology, Cheikh Anta Diop University, Avenue Cheikh Anta Diop, BP 5005 Fann, Dakar, Senegal; 2 National Malaria Control Program (NMCP), Rue Aimé Césaire, Fann Résidence, Dakar, Senegal

**Keywords:** External quality assessment, EQA, Microscopy, Diagnosis, Malaria, Senegal

## Abstract

**Background:**

In developing countries, malaria diagnosis relies on microscopy and rapid diagnostic tests. In Senegal, national malaria control program (NMCP) regularly conducts supervisory visits in health services where malaria microscopy is performed. In this study, expert microscopists assessed the performance of laboratory technicians in malaria microscopy.

**Methods:**

The present external quality assessment (EQA) was conducted in three different areas of malaria transmission. Participants were laboratory technicians previously trained by NMCP on malaria microscopy. Stored read slides were randomly collected for blinded re-checking by expert microscopists. At the same time a set of 8 slides (3 positive *P. falciparum* and 5 negative slides) were submitted to participants for proficiency testing. Microscopists performance were evaluated on the basis of the errors rates on slide reading—high false positive (HFP), high false negative (HFN), low false positive (LFP) and low false negative (LFN)—and the calculation of their sensitivities and specificities relative to expert microscopy. Data were entered and analysed using Microsoft Excel software.

**Results:**

A total of 450 stored slides were collected from 17 laboratories for re-checking. Eight laboratories scored 100% of correct reading. Only one major error was recorded (HFP). Six laboratories recorded LFN results: Borrelia, *P. ovale*, and low parasite densities (95 and 155 p/μl) were missed. Two *P. falciparum* slides were misidentified as *P. malariae* and one *P. ovale* slide as *P. vivax*. The overall sensitivities and specificities for all participants against expert microscopists were 97.8 and 98.2% respectively; Sensitivities and specificities of hospital microscopists (96.7 and 98.9%) were statistically similar to those of health centre microscopists (98.5 and 97.8% respectively) (p = 0.3993 and p = 0.9412 respectively). Overall, a very good agreement was noted with kappa value of 0.96 (CI_95%_ 93.4–98.6%) relative to expert microscopy. Proficiency testing showed that among the 17 participants, 11 laboratories scored 100% of correct reading. Three LFN and four LFP results were recorded respectively. The *P. falciparum* slide with Maurer dots was misidentified as *P. ovale* in 1 centre and the same slide was misread as *P. vivax* in another centre; No major error (HFP or HFN) was noted.

**Conclusion:**

EQA of malaria microscopy showed an overall good performance especially regarding *P. falciparum* detection. However, efforts need to be made addressing the ability to detect non-falciparum species and others endemic blood pathogens such as *Borrelia*. The further NMCP training sessions and evaluations should consider those aspects to expect high quality-assured capacity for malaria microscopy.

## Background

Despite the development of new diagnostic tools, microscopy still remains the gold standard method for malaria diagnosis [1]. Microscopy of thick and thin smears is relatively simple and inexpensive, and particularly suitable to rapidly diagnose malaria. Moreover, microscopy allows species identification and parasite count [2], and it is also suitable to diagnose infections with other pathogens that may be detected microscopically in routinely Giemsa-stained smears, such as spirochetes of *Borrelia*, which are common in Senegal [3]. However, reliability of malaria microscopy is highly dependent on competency of laboratory staff [2]. As a result, reinforcing strategies to promote good quality of microscopy-based malaria diagnosis has been gaining an increased interest in the global malaria agenda [4, 5].

In order to evaluate laboratory performance on malaria microscopy, WHO recognize the need to establish comprehensive external quality assessment (EQA) system [5]. However, little research on the evaluation of EQA systems in malaria microscopy has been conducted in Senegal. National Malaria Control Programs (NMCP) has defined a procedure to ensure good quality of malaria microscopy. This study aimed to evaluate the performances in malaria microscopy among laboratory technicians in Senegal.

## Methods

### Description

In Senegal, EQA procedures have been established by NMCP and regularly supervisory visits are conducted in all levels of malaria laboratory services. Also, NMCP organize periodic training towards lab technicians in conventional methods of malaria diagnosis. During the training sessions, participant laboratories are notified to store read slides in two separate provided boxes (Negative and Positive) for further quality control re-check. During the NMCP supervisory visits, senior microscopists assess the competence of laboratory technicians in performing thick and thin smear, reading slides, microscope maintenance and reagents management.

The collected results are analysed and reported to the NMCP, which conducts corrective actions for quality improvement.

### Sites and participants

**Fig. 1:**
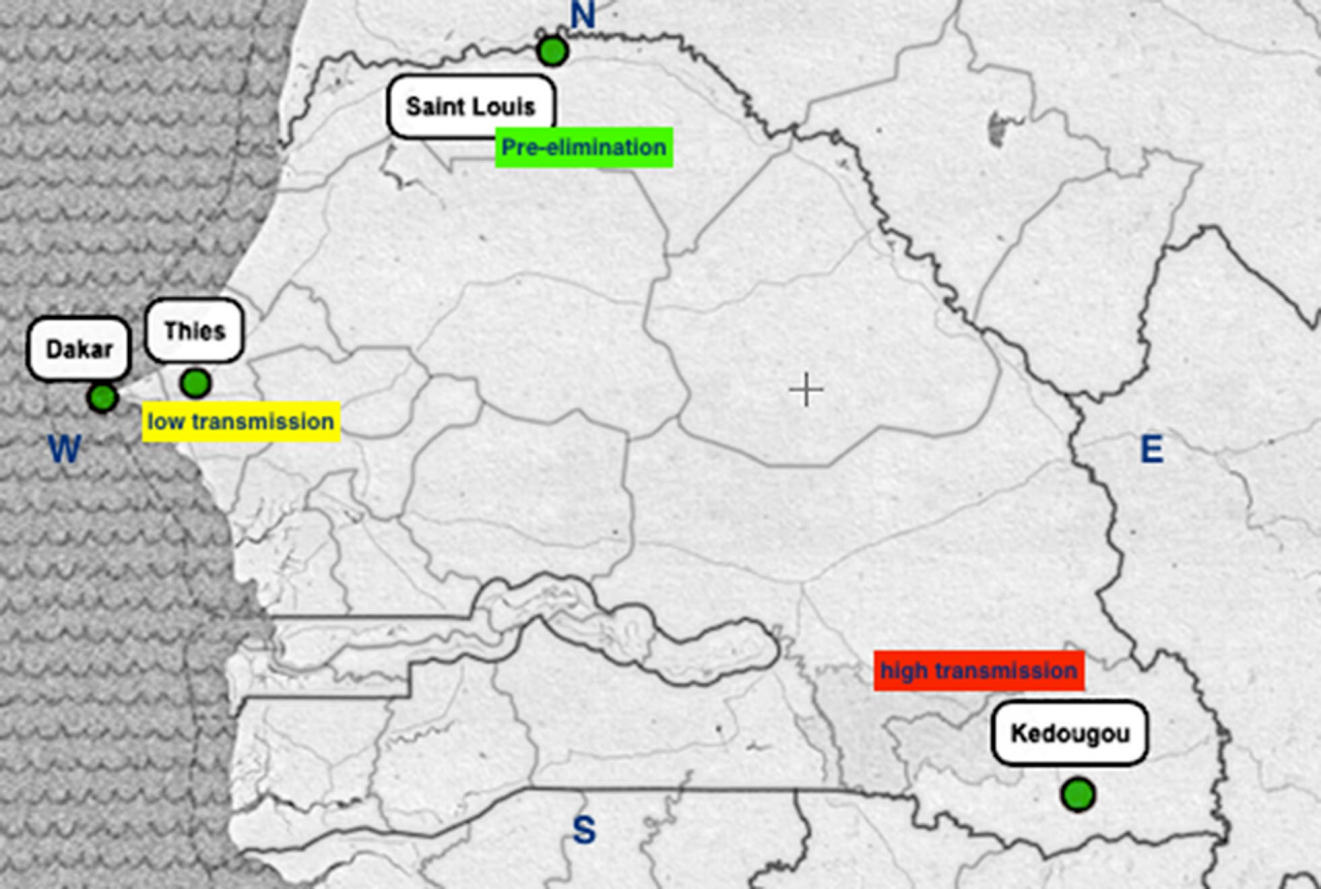
Malaria epidemiological strata with sites where supervision was conducted; 7 hospital laboratories: Dakar (3), Thies (1) Saint Louis (2), Kedougou (1); 11 health centre laboratories: Dakar (2), Thies (2), Saint Louis (4), Kedougou (3)

A cross-sectional study was conducted in three different strata of malaria distribution: (1) Saint Louis in the north (pre-elimination stage), (2) Dakar and Thies in the west centre (low prevalence) and (3) Kedougou in the south (high prevalence). Seventeen laboratories (seven hospitals and eleven health centres) participated (Fig. 1); Participants were lab technicians previously trained during training sessions conducted by NMCP.

### On-site evaluation

A supervisory checklist was submitted to participants to assess the status of infrastructure, availability of comprehensive standard operating procedure (SOP), appropriate use of reagents and equipment, maintenance of microscope, laboratory safety, training related to malaria microscopy, data and supply management, internal quality control (QC) procedure.

Also, supervisors examined that the slides are stored according to the appropriate SOPs and that the results are reported in a register with the following mentions: species identification (instead of mentioning “Positive results”), parasite stages (young trophozoites, mature trophozoites, schizonts and gametocytes), and assessment of parasite density.

### Slides rechecking

Supervisors randomly selected 30 slides from each participant laboratory (15 samples of the smears from each negative and positive boxes where slides were stored). Seventeen laboratories were supervised. Two health centres were not able to store their read slides as required by NMCP so they were not included on the rechecking evaluation. Thus, a total of 450 slides (225 declared positives and 225 declared negatives) were collected from the 15 remaining laboratories. The selected smears were sent to the national reference laboratory (NRL) where they are blindly re-examined by two WHO level 1 malaria microscopists. Slides yielding discrepant results between two experts are resolved by a third expert microscopist.

### Proficiency testing

**Fig. 2:**
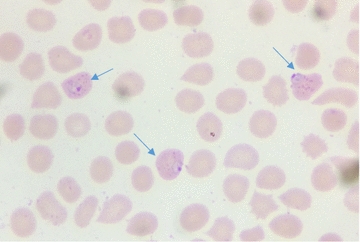
Trophozoites of *P. falciparum* with specific Maurer dots inside the infected red blood cells, Giemsa stained thin smear (×1000)

Along with collecting stored slides, a set of eight reference slides was distributed to all involved participant microscopists: slide (1) *Plasmodium falciparum* moderate parasite density (28,000 p/μl) with Maurer dots (Fig. 2), slide (2) *P. falciparum* low parasite density (192 p/μl), slide (3) *P. falciparum* gametocyte and trophozoites with high parasite density and slides 4–8) five slides with no parasites.

The set of submitted slides consisted of reference slides from national malaria slide bank. The malaria slide bank is certified by WHO and is hosted in the Laboratory of Parasitology-Mycology of Aristide Le Dantec Hospital which is the national reference laboratory (NRL) for the Senegal NMCP. The NRL had six expert microscopists during past external competency assessment of malaria microscopy (ECAMM) courses.

### Data analysis

The results of the EQA system were evaluated using the following indicators: major errors [high false-positive (HFP), high false-negative (HFN)], minor errors [low false-positive (LFP), low false-negative (LFN)] and misidentification; these indicators were defined depending on the modality of the evaluation:

#### Slides rechecking

LFP was defined as only one false positive is noted among the ten collected labelled negative by participants and HFP when there was more than one false positive. LFN was considered if the following slides were missed by participants among the ten labelled positive slides: parasite density less than 200 p/μl, spirochetes, non-falciparum species. HFN was considered when slides with parasite density more than 200 was missed.

Misidentification refers to true positive slides that were detected positive by participants but species identification was not correct.

#### Proficiency testing

LFP was defined when only one submitted reference negative slide is read positive by the participant and HFP when there were more than one false positive among the five negative slide. LFN was noted if only the slide with *P. falciparum* low parasite density (192 p/μl) was missed and HFN when slide with medium and high parasite density was missed by the participant.

Misidentification refers to reference positive slides that were detected positive by participants but species identification was not correct.

Sensitivity and specificity were calculated to assess the microscopy reading performance of the participants relative to the expert microscopy. Data were analysed using Excel (version 15.27). kappa statistic test was used to assess the magnitude of agreement between expert microscopists and participants. Exact Chi square analysis was used to determine the significance differences between sensitivities, specificities, negative predictive value (NPV) and positive predictive value (PPV). The p-values less than 0.05 were considered as statistical significance.

### Ethical consideration

This study did not require informed consent from participants as no human subjects were involved. Ethical approval was obtained from Senegalese National Ethic Committee of Ministry of Health.

## Results

### On-site evaluation

Among the 17 participants, 14 laboratories have posted smear & staining procedure and parasite counting method and 15 laboratories have arranged read slides in boxes accordingly to NMCP procedure. Two laboratories were using expired Giemsa reagent. Internal quality control (QC) was followed only by 1 laboratory.

All laboratories were using standardized laboratory register provided by NMCP; however, only 5 reported their results with complete information on the different sections of the registers; concerning the positive slide reporting results, 5 laboratories mentioned “Positive” without giving any details, 7 laboratories reported the specie “*Plasmodium falciparum*” without the differentiation stage. None laboratory mentioned the presence of gametocytes nor schizonts, although those stages were seen during re-examination process. Three participants did not report the parasite counting on their registers.

All reagents related to malaria microscopy were supplied directly through NMCP. All participant facilities were suitable for standards of infrastructure, safety and waste disposal practices. Microscope maintenance was respected by all laboratories.

### Proficiency testing

Among the 17 participants, 11 laboratories scored 100% as they correctly detected negative and positive reference slides. Three laboratories (one hospital and two health centres) misread negative slides as positive (LFN errors), four laboratories (one hospital and three health centres) misread low parasite density slide as negative (LFP errors); Two centres misread the *P. falciparum* slide with Maurer dots (Fig. 2) as *P. ovale* while one centre misread it as *P. vivax*; No major errors (HFP or HFN) were noted (Table 1).

**Table 1:** Reported errors from participants on panel testing slides

Participants	Slide 1	Slide 2	Slide 3	Slide 4–8	Total errors
Hospital 1	Negative			*P. falciparum*	2
Hospital 5		*P. ovale*			1
HC 1	Negative			*P. falciparum*	2
HC 5		*P. ovale*			1
HC 8	Negative	*P. vivax*		*P. falciparum*	3
HC 10	Negative				1
Total errors	4	3	0	3	10

Slide 1: *P. falciparum*, 192 p/μl

Slide 2: *P. falciparum*, 28,000 parasites with Maurer dots (see Fig. 1)

Slide 3: *P. falciparum*, with both trophozoites and gametocytes

Slides 4,5,6,7 and 8: No parasites

### Cross-checking of slides

**Fig. 3:**
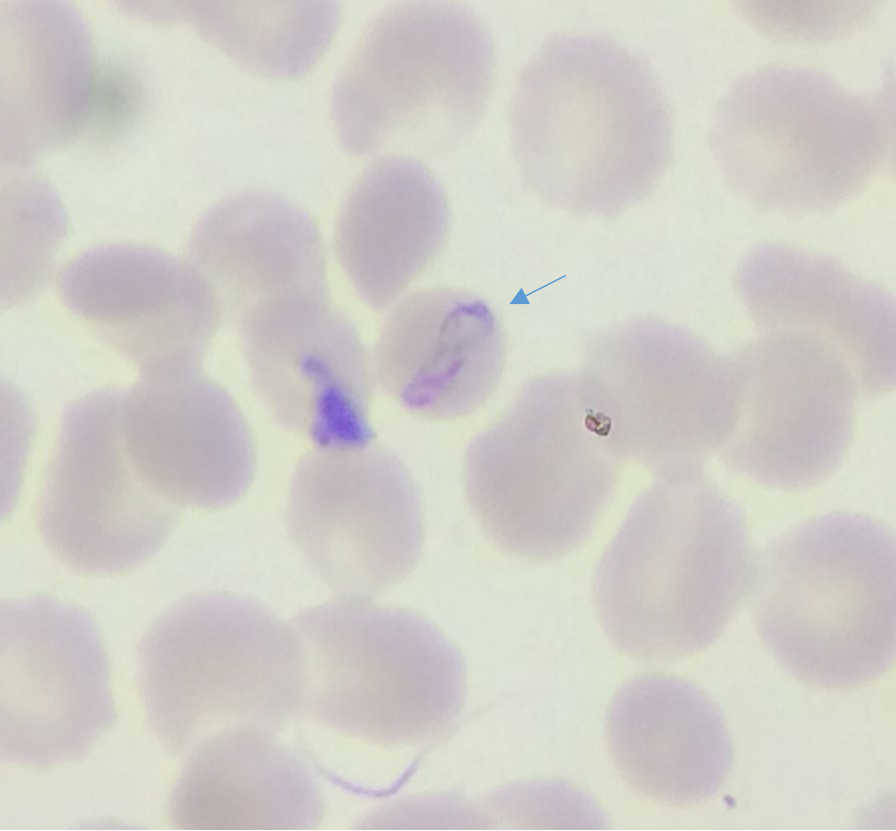
*P. falciparum* trophozoite presenting an equatorial-like band in Giemsa stained thin smear (×1000)

Overall, 450 stored and collected slides from 15 participant laboratories were rechecked by WHO expert microscopists. Two health centres were excluded for this evaluation as they could not store their read slides as required by NMCP;

Eight laboratories achieved zero error. Only one major error was recorded as one health centre misread 2 negative slides as positive (HFP). Six laboratories recorded LFN results with the following positive slides misread as negative: spirochetes of Borrelia (missed by two hospital microscopists), *P. ovale* (missed by two health centres), and two slides with low parasite density of *P. falciparum* (missed by two health centres); the low parasite densities that were missed were 95 and 155 p/μl. Three laboratories detected parasites on true positive slides but species identification was incorrect: two hospitals misidentified *P. falciparum* slides as *P. malariae* and one health centre participant misidentified *P. ovale* slide as *P. vivax* (Tables 2, 3). When re-examining misidentified *P. falciparum* slides, expert microscopists observed “band-like forms” of trophozoites similar to that of *P. malariae* (Fig. 3); however, the absence of malarial pigments associated with high parasitaemia and the not-variegated aspect of the smear, were indicative of atypical *P. falciparum*.

**Table 2:** Errors reported during cross-checking of read slides

Participants laboratories	Errors types					Total errors
	HFP	HFN	LFP	LFN^a^	Misidentification^a^	
Hospital 1	0	0	1	1	3	5
Hospital 2	0	0	0	1	0	1
Hospital 3	0	0	0	0	0	
Hospital 4	0	0	0	1	1	2
Hospital 5	0	0	0	0	0	
Hospital 6	0	0	0	0	0	
HC 1	0	0	0	0	0	
HC 2	2	0	0	0	0	2
HC 3	0	0	0	0	0	
HC 4	0	0	0	0	0	
HC 5	0	0	1	0	0	1
HC 6	0	0	0	0	1	1
HC 7	0	0	0	0	0	
HC 8	0	0	0	0	0	
HC 9	*NA*	*NA*	*NA*	*NA*	*NA*	
HC 10	*NA*	*NA*	*NA*	*NA*	*NA*	
HC 11	0	0	0	2	0	2

HC9 and HC10 were not involved as they could not store the required number of slides

^a^See details on Table 3

**Table 3:** Results of missed slides and misidentified slides when controlled by expert microscopists

	Results of participants	Results of controllers
	Misidentification	
Hospital 1	*P. malariae*	*P. falciparum*
Hospital 4	*P. vivax*	*P. ovale*
	LFN	
Hospital 1	Negative	Spirochetes
Hospital 2	Negative	Spirochetes
Hospital 4	Negative	*P. falciparum*, 95 p/μl
HC 4	Negative	*P. ovale*
HC 11	Negative	*P. ovale*
HC 11	Negative	*P. falciparum*, 155 p/μl

The overall sensitivities and specificities for all participants against expert microscopists were 97.8 and 98.2% respectively; Sensitivities and specificities of hospital microscopists were 96.7 and 98.9% respectively while those of health centre microscopists were 98.5 and 97.8% respectively (Table 4). Overall, very good agreement was noted with kappa value of 0.96 (CI_95%_ 93.4–98.6%). No statistically significant differences of sensitivities and specificities were found between hospital and health centre microscopists (p = 0.3993 and p = 0.9412 respectively).

**Table 4:** Sensitivities, specificities, PPV and NPV of participant microscopists against expert microscopists

		Controllers		Total
		Positive	Negative	
Hospital technicians	Positive	89	1	90
	Negative	3	87	90
	Total	92	88	180
Sensitivity (%)	96.7			
Specificity (%)	98.9			
PPV (%)	98.9			
NPV (%)	96.7			
Health centers technicians	Positive	132	3	135
	Negative	2	133	135
	Total	134	136	270
Sensitivity (%)	98.5			
Specificity (%)	97.8			
PPV (%)	97.8			
NPV (%)	*98.5*			

## Discussion

In Senegal, malaria transmission usually occurs during the rainy season (June–December) and the beginning of the dry season (January). This period corresponds to development of high density of vector populations. The prevalence of parasitaemia at the nation level have decreased from 5.9% in 2008 to 2.8% in 2013 in relationship to policies carried out by NMCP. However, high disparities of malaria prevalence exist between endemic regions. Mainly, three different epidemiological profiles have been identified [6] in which three human infecting species, *P. falciparum*, *P. malariae* and *P. ovale* are known to circulate [7]. Low values of parasitaemia prevalence are concentrated in the northern Senegal, particularly in the region of Saint-Louis, Louga and Matam. Malaria risk increases in some areas of central Senegal and reaches the highest values in the southern (Kolda, Tambacounda and Kedougou) [8, 9]. In the west centre, the parasite prevalence is about 1% in Dakar and Thies [6]. The urban malaria burden is concentrated in the cities where the anopheles vector density is very low [8].

Malaria diagnosis is one of the key strategy for effective malaria control and elimination programs. Microscopy still remains the mainstay of malaria diagnosis in endemic countries [1]. Maintaining a quality-assured microscopy service is a major challenge [10]. The quality of microscopy-based malaria diagnosis has been questioned in several studies [11–14].

This study showed that malaria microscopy in Senegal had shown an overall low total errors during this supervisory visit, particularly when addressing *P. falciparum* detection. Senegal NMCP arranged regular training system, an effective supply management and regular periodic supervisions visits with on-site corrective actions; this most probably contributed to achieve this good performance.

In Senegal, for many years the NMCP targeted especially falciparum malaria to fight against malaria. Yet, it is well known that non-falciparum species are circulating in the country [15–17]. Moreover, WHO has recommended to integrate in the EQA other local pathogens that may be detected microscopically. In Senegal, *Borrelia crocidurae*, a bacterial spirochete, is a major cause of fever (thick-borne relapsing fever). The bacteria may be observed by experienced microscopists when examining routinely malaria thick and thin smears [18]. As a result, slides positive with spirochetes were included in the NRL malaria slide bank with the aim to train and evaluate malaria microscopists on this.

In this evaluation, the *P. falciparum* slide with Maurer dots was introduced on purpose to assess the skills of microscopists to recognize such granulations. In fact, most of the time in peripheral laboratories and even in hospital laboratories, Giemsa stain is diluted with tap water; in this condition, *P. falciparum* Maurer dots do not appear and lab technicians may be unfamiliar with those findings when slides are stained appropriately using buffered solution.

In endemic African countries it has been reported misidentifications of *Plasmodium* species, particularly for the non-falciparum species [11]. Difficulties to distinguish the non-falciparum species are common, especially for those with low prevalence [12]. Moreover, the NMCP tends to neglect non-falciparum malaria due to its less severe clinical picture compared to falciparum malaria [15].

The non-identification of the stages of *P. falciparum* was a real challenge. In fact, presence of gametocytes can be an indicator of therapeutic failure and even resistance to some antimalarial drugs [19]. Moreover, in low transmission areas where malaria elimination is planned, the proportion of individuals carrying gametocytes is critical to the maintain the transmission of the disease.

In Senegal, during the previous NMCP training sessions and proficiency testing, only *P. falciparum* slides were used to train malaria microscopists and to evaluate their performance. This supervisory report led the NMCP to consider revisions on the training format for the further sessions. For the first time, the training is supported by a validated reference slide set. Currently, the NRL through the malaria slide bank is able to provide reference slides for all non-falciparum species, falciparum low parasite densities, atypical trophozoites of *P. falciparum* and spirochetes of *Borrelia*.

Also, the NMCP initiated a program for microscopists to undergo WHO external competency assessment on malaria microscopy (ECAMM) courses; the first round allowed to identify a core group of microscopists (WHO level 1 and 2). Some of those leading microscopists have been selected to undergo training-of-facilitators courses.

Thus, the next EQA should include more restringing requirements and this study could be considered as the baseline. Microscopists in the country will be assessed and certified in a national competence assessment (NCA) system. The protocol for these assessment and certification will include parasite density determination, non-falciparum species identification, falciparum low parasite density detection, atypical trophozoites of *P. falciparum* recognition and spirochetes of *Borrelia* detection. It is expected, with this program, to increase trust among clinicians so they will prescribe appropriately antimalarial drugs [20]. A preceded retraining program is planned before evaluation. In fact, refresher training as well as adequate equipment and reagents, good working conditions, motivated and qualified personnel are known to improve microscopists skills [12, 14, 21].

In Senegal, according to NMCP procedure for cross-checking slides, the number of randomly selected slides is higher than that of WHO recommendations [22]. The advantage of a large number of taken slides is to increase the power of the study to detect slide reading errors; this is particularly relevant in regions striving to eliminate malaria, such as in north Senegal.

## Conclusion

In Senegal, the performance quality of malaria microscopy is relatively satisfactory especially when addressing *P. falciparum* detection. However, non-falciparum species and blood bacterial such as Borrelia are likely to be missed when performing malaria microscopy. Another approaches are recommended to be adapted by future trainings and evaluations on microscopic laboratories.

## Abbreviations

ECAMM: external competency assessment of malaria microscopists
EQA: external quality assessment
HC: health centre
HFN: high false-negative
HFP: high false-positive
LFN: low false-negative
LFP: low false-positive
NCA: national competence assessment
NMCP: national malaria control program
NPV: negative predictive value
NRL: national reference laboratory
PPV: positive predictive value
QC: quality control
RDT: rapid diagnostic test
SOP: standard operating procedures
WHO: World Health Organization
